# The Multitalented MEDIATOR25

**DOI:** 10.3389/fpls.2017.00999

**Published:** 2017-06-12

**Authors:** Kemal Kazan

**Affiliations:** ^1^Commonwealth Scientific and Industrial Research Organisation Agriculture and Food, BrisbaneQLD, Australia; ^2^Queensland Alliance for Agriculture and Food Innovation, Queensland Bioscience Precinct, The University of Queensland, BrisbaneQLD, Australia

**Keywords:** ABA signaling, abiotic stress, auxin signaling, ethylene signaling, jasmonate signaling, Mediator complex, pathogen defense, PFT1

## Abstract

The multi-subunit Mediator complex, which links DNA-bound transcription factors to RNA Pol II during transcription, is an essential regulator of gene expression in all eukaryotes. Individual subunits of the Mediator complex integrate numerous endogenous and exogenous signals. In this paper, diverse regulatory functions performed by MEDIATOR25 (MED25), one of the subunits of the plant Mediator complex are reviewed. MED25 was first identified as a regulator of flowering time and named PHYTOCHROME AND FLOWERING TIME1 (PFT1). Since then, MED25 has been implicated in a range of other plant functions that vary from hormone signaling (JA, ABA, ethylene, and IAA) to biotic and abiotic stress tolerance and plant development. MED25 physically interacts with transcriptional activators (e.g., AP2/ERFs, MYCs, and ARFs), repressors (e.g., JAZs and Aux/IAAs), and other Mediator subunits (MED13 and MED16). In addition, various genetic and epigenetic interactions involving MED25 have been reported. These features make MED25 one of the most multifunctional Mediator subunits and provide new insights into the transcriptional control of gene expression in plants.

## Introduction

Transcriptional regulation of gene expression in eukaryotes is one of the most intricate cellular processes involving many proteins and protein complexes working in coordination. Among the transcriptional regulators, transcription factors (TFs) play essential roles in activating or repressing transcription in response to endogenous and exogenous cues. However, TFs do not directly interact with RNA Pol II, the multiprotein enzyme complex involved in transcription. Instead, TFs directly bind to specific *cis*-acting/enhancer sequences found in the promoter regions of target genes through their DNA-binding domains and recruit other components of the transcriptional machinery such as coactivators, corepressors, and chromatin modifying enzymes (e.g., histone acetyltransferases and histone deacetylases). TFs also recruit via their *trans*-activation domains (TADs), the Mediator multiprotein complex, which bridges TFs to the RNA Pol II during transcription. In addition to its role in activator-mediated transcription, Mediator stimulates basal transcription and is involved in transcriptional silencing (Karijolich and Hampsey, 2012).

### Mediator Complex and Transcriptional Control of Gene Expression in Eukaryotes

Mediator has been first discovered in yeast (*Saccharomyces cerevisiae*), which contains 25 subunits, while approximately 31 and 34 subunits are found in mammals and plants, respectively (reviewed in Mathur et al., 2011; Allen and Taatjes, 2015; Samanta and Thakur, 2015). The Mediator complex is organized into four modules; the Head, Middle, Tail, together known as the core Mediator, and CYCLIN-DEPENDENT KINASE 8 (CDK8), which is reversibly associated with Mediator. In yeast and Metazoan species, CDK8 modulates transcription by phosphorylating TFs and influencing RNA Pol II-Mediator interactions (Poss et al., 2013; Tsai et al., 2013; Nemet et al., 2014). The Tail module of the Mediator complex interacts with the DNA-bound TFs while the Head module interacts with RNA Pol II and may also be involved in basal or activator-independent transcription. The Middle module provides the flexibility required for this large protein complex to exhibit necessary conformational changes in response to RNA Pol II binding (Karijolich and Hampsey, 2012).

Mediator can modulate RNA Pol II transcription by influencing the assembly of the pre-initiation complex (PIC), Pol II pausing, elongation and re-initiation and chromatin architecture (Allen and Taatjes, 2015). It is now well-established that the Mediator complex is involved in both transcriptional activation and repression. Indeed, Mediator can interact with both transcriptional activators and also repressors (Dolan and Chapple, 2017). In addition to its activator roles, the CDK8 kinase module is involved in repressing transcription by binding to the Tail module and blocking the interaction of Mediator with RNA Pol II (Conaway and Conaway, 2011).

In this review, diverse functions performed by MED25, one of the relatively well-conserved subunits of the Mediator complex, will be reviewed. Please see recent reviews for additional information on the plant Mediator complex (Buendía-Monreal and Gillmor, 2016; Yang et al., 2016; Dolan and Chapple, 2017).

### MED25 and the Plant Mediator Complex

The discovery of MED25 in plants was somewhat serendipitous. First, screening of mutagenized Arabidopsis populations identified the recessive *pft1* (*phytochrome and flowering time 1*) mutant that showed a late flowering phenotype under suboptimal light conditions. This suggested that the mutated gene regulates shade-avoidance (rapid extension of stem and accelerated flowering in plants grown under dense canopies) (Cerdán and Chory, 2003). Subsequent genetic analyses placed PFT1 downstream from phyB and upstream from FLOWERING TIME (FT), a positive regulator of flowering. The cloning of the mutated gene in the *pft1* mutant has revealed that PFT1 is a nuclear protein with similarity to some transcriptional activators from animals (Cerdán and Chory, 2003). However, it was not realized until the biochemical purification of the Mediator complex from plants that *PFT1* encodes MED25 (Bäckström et al., 2007). As explained in detail below, since then, the *med25* mutant has been independently identified in different forward screens (Zheng et al., 2006; Seguela-Arnaud et al., 2015) and a number of functions regulated by PFT1/MED25 characterized.

### Functional Domains of MED25

The yeast does not contain a MED25 homolog while a single gene encoding MED25 is found in plants (Kidd et al., 2011). The location of MED25 within the Mediator complex is unknown. However, the finding that MED25 interacts with a number of TFs (see below) and also with MED16, a Tail module subunit, suggests that MED25 is most likely associated with the Tail module of the Mediator complex (reviewed by Kidd et al., 2011; Yang et al., 2014).

**FIGURE 1:**
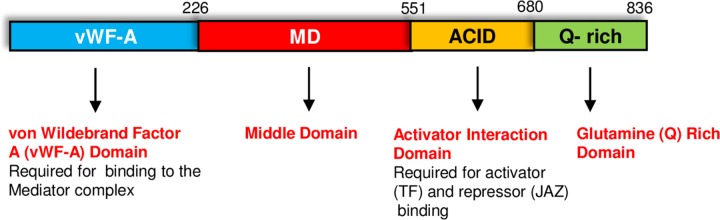
The MED25 protein contains multiple domains. The Activator Interaction Domain (ACID) region interacts with MYC and other transcriptional regulators, while the conserved von Wildebrand Factor A (vWF-A) domain is required for the interaction of MED25 with the Mediator complex. Please see text for additional details.

The MED25 protein contains multiple domains that perform specific functions (**Figure 1**). The conserved von Willebrand Factor Type A (vWF-A) domain located at the amino terminus of MED25 is required for its interaction with the Mediator complex via the Tail subunit MED16 (Yang et al., 2014). The ACID (Activator Interacting Domain) region is required for the interaction of MED25 with transcriptional activators such as AP2/ERF and MYC TFs as well as suppressors such as JAZ proteins (Çevik et al., 2012; Chen et al., 2012; Zhang et al., 2015). VP16, a TF from the Herpes simplex virus with strong transcription activation activity in plants also binds to MED25-ACID through its TAD (Yang et al., 2004). MED25 also contains a conserved glutamine (Q) rich poly-track in its C-terminus, possibly involved in transcriptional activation (Cerdán and Chory, 2003; Bäckström et al., 2007; Elfving et al., 2011). The presence or absence of this Q-rich region or its length influences flowering-associated functions of MED25 (Rival et al., 2014). The alternative splicing of the Q-rich region of *MED25*, which generates two different isoforms, is evolutionary conserved and potentially contributes to diverse functions performed by this protein (Rival et al., 2014). As explained in detail below, MED25 does not directly interact with RNA pol II but activates or represses transcription by interacting with activators or repressors (Kidd et al., 2009; Yang et al., 2016; Dolan and Chapple, 2017).

## Med25, A Master Regulator of Plant Hormone Signaling

As reviewed below, recent findings show that MED25 regulates multiple phytohormone signaling pathways and phytohormone crosstalk.

### MED25 and JA Signaling

The plant hormone JA regulates responses to biotic and abiotic stresses (Kazan, 2015). Under low hormone levels (i.e., no stress), JA responses are suppressed by JAZ proteins through their interactions with MYC TFs and the co-repressor TPL recruited either directly through JAZ repressor EAR (Ethylene-responsive element binding factor-associated amphiphilic repression) motifs (Kazan, 2006) or an accessory protein called NINJA, which also contains an EAR motif (Kazan and Manners, 2012, 2013; Wasternack and Song, 2016). JAs produced under stress conditions are sensed by the COI1-JAZ co-receptor complex. This leads to the destruction of JAZ repressors and the relief of repression on MYC TFs, which then regulate diverse JA responses (Kazan and Manners, 2013). However, until the discovery of MED25, how MYC TFs and other JA responsive TFs recruit the Mediator complex, had remained enigmatic (Kidd et al., 2009).

The roles of PFT1/MED25 in JA signaling was first identified in a genome-wide analysis of Arabidopsis TFs as a putative TF responsive to the fungal pathogen *Alternaria brassicicola* and methyl JA (McGrath et al., 2005). The Arabidopsis *pft1/med25* mutant shows attenuated expression of JA responsive defense genes and altered resistance to necrotrophic pathogens, suggesting that MED25 controls an essential point within the JA pathway (Kidd et al., 2009). In addition, the *bestatin resistant 6* (*ber6*) mutant, which shows sensitivity to root inhibition by bestatin, a strong chemical activator of the JA signaling pathway, was found to be allelic to *med25* (Zheng et al., 2006).

### MED25 Regulates JA Signaling by Interacting with Activators and Repressors

The discovery of physical interactions between MED25 and various JA-associated TFs has provided further evidence about the role of MED25 and JA signaling. First, a high throughput yeast two hybrid (Y2H) screening of 1589 Arabidopsis TFs from 62 different families using the conserved ACID region of MED25 as bait has identified eight MED25-interacting TFs, including four AP2/ERFs belonging to Group IX of the AP2/ERF family (Ou et al., 2011) implicated in JA signaling and pathogen resistance (McGrath et al., 2005). Five of these TFs could bind to the GCC box promoter element (Ou et al., 2011), which confers JA responsiveness (Brown et al., 2003). Subsequently, other JA responsive TFs, including ORA59 (OCTADECANOID-RESPONSIVE ARABIDOPSIS AP2/ERF59) and ERF1 (ETHYLENE RESPONSE FACTOR1) as well as the master regulator MYC2 and related MYC TFs MYC3 and MYC4 have been shown to be interacting with MED25 (Çevik et al., 2012; Chen et al., 2012). The conserved EDLL motif (Tiwari et al., 2012) present in the carboxyl-terminal region of AP2/ERFs such as ERF1, ERF15, and TDR1/ERF98, seems to be important for the interaction of these AP2/ERFs with the ACID region of MED25 (Çevik et al., 2012). MED25 seems to genetically act downstream from ORA59 and ERF1 and is required for JA-mediated activation of the pathogen defense genes such as *PDF1.2*. In addition, MED25 is required for MYC2-mediated activation of insect defense gene *VSP* and MYC2-dependent suppression of *PDF1.2* during JA signaling (Kidd et al., 2009, 2010; Çevik et al., 2012).

Similarly to MYC TFs, JAZ repressors such as JAZ9 interact with MED25 through their conserved JAS (JA Associated) domains (Zhang et al., 2015). So, it appears that both repressors (e.g., JAZs) and activators (e.g., MYCs) compete for binding to MED25 during JA signaling. Therefore, interfering with the MYC-MED25 interaction seems to be one of the mechanisms employed by JAZ proteins to suppress JA-responsive gene expression (Zhang et al., 2015, 2017).

**FIGURE 2:**
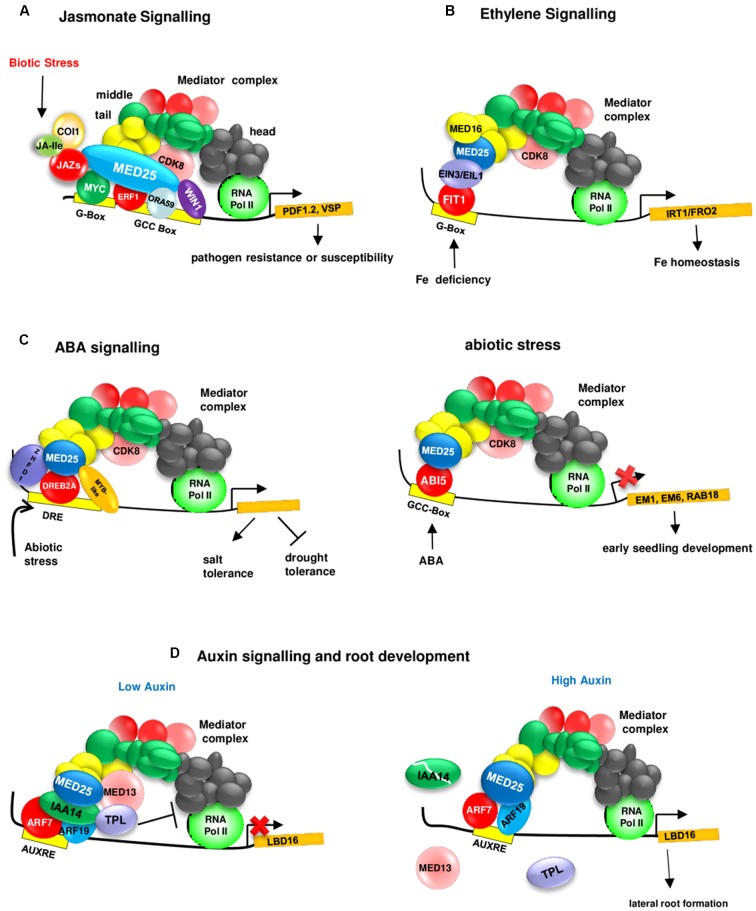
MED25 regulates plant hormone signaling through its interaction with different transcriptional regulators. **(A)** MED25 is a master regulator of JA signaling. MED25 interacts with JAZ proteins acting as repressors of JA signaling, multiple activators (e.g., MYCs that bind to the G-box and AP2/ERFs that bind to the GCC box) and recruits RNA polymerase II to the promoters of JA-responsive genes such as (*PDF1.2* and *VSP1*). JA-Ile:Jasmonate Isoleucyl. Please see text for additional details. **(B)** MED25 is a regulator of ethylene signaling during nutrient deficiency responses. MED25 by interacting with the ET signaling components EIN3 and EIL1 and the Mediator complex subunit MED16 plays a role in transmitting iron deficiency signals to RNA Pol II for the activation of genes (e.g., *IRT1*/*FRO2*) involved in iron homeostasis. Please see text for additional details. MED16, MEDIATOR 16; EIN3, ETHYLENE INSENSITIVE 3; EIL1, ETHYLENE INSENSITIVE 3 LIKE 1. **(C)** MED25 is a regulator of ABA (left image) and abiotic stress (right image) signaling. MED25 interacts with the ABI5 TF and negatively regulates the expression of ABA-responsive genes (*EM1*, *EM6*, and *RAB18*) involved in early seedling development. MED25 interacts with abiotic stress responsive TFs (such as DREB2A, which binds to the DRE box) and positively and negatively regulates salt and drought tolerance. **(D)** MED25 is a regulator of auxin signaling. Under low auxin levels (left image), a suppressive complex involving AUX/IAA14, TPL, and MED13 form a suppressive complex at the promoter (right image) of *LBD19* encoding a TF involved in lateral root development to inhibit the interaction between the core Mediator complex and RNA Pol II. Auxin leads to the destruction of this complex and ARF7 and ARF19, positive regulators of auxin signaling interact with MED25 and recruit RNA Pol II to the *LBD16* promoter.

MED25 also interacts with CDK8 (Zhu et al., 2014; **Figure 2** and **Table 1**). Interestingly, similarly to MED25, CDK8 positively regulates JA-responsive defense gene expression (e.g., *PDF1.2*) and resistance to *A. brassicicola*. However, in contrast to MED25, which positively regulates resistance to *Botrytis cinerea* (Kidd et al., 2009), CDK8 negatively regulates resistance to this pathogen (Zhu et al., 2014). Furthermore, both MED25 and CDK8 interact with WIN1 (WAX INDUCER 1) (Zhu et al., 2014), an AP2/ERF TF that regulates cuticle development and plant defense (Sela et al., 2013). Together, these findings suggest that MED25 and CDK8 perform overlapping functions within the JA signaling.

**Table 1:** MED25 interacting proteins.

MED25 interacting protein	MED25 interaction domain	Protein family	Function	Reference
ABI5 (ABA INSENSITIVE 5) (AT2G36270)	ACID	Basic leucine zipper TF	ABA signaling	Chen et al., 2012
Basic leucine-zipper 8 (AT1G68880)	ACID	Basic leucine zipper TF	Unknown	Çevik et al., 2012
MBR1 (MED25 BINDING RING-H2 PROTEIN 1 (AT2G15530)	vWF-A domain	RING/U-box domain-containing protein	Degrades MED25 and promotes flowering	Iñigo et al., 2012b
MBR2, MED25 BINDING RING-H2 PROTEIN 2 (AT4G34040)	vWF-A domain	RING/U-box superfamily protein	Degrades MED25 and promotes flowering	Iñigo et al., 2012b
ERF1, ETHYLENE RESPONSE FACTOR1 (AT3G23240)	ACID	AP2/ERF TF	JA and ET signaling disease resistance and drought tolerance	Ou et al., 2011; Çevik et al., 2012
ORA59 (AT1G06160)	ACID	AP2/ERF TF	JA and ET signaling	Çevik et al., 2012
TDR1 (AT3G23230)	ACID	AP2/ERF TF	Biotic stress	Çevik et al., 2012
ERF15 (AT2G31230)	ACID	AP2/ERF TF	Biotic and abiotic stress	Çevik et al., 2012
RAP2.2 (AT3G14230)	ACID	AP2/ERF TF	Biotic and abiotic stress	Ou et al., 2011
ERF109/RRTF1 (redox responsive TF 1) (AT4G34410)	ACID	AP2/ERF TF	Crosstalk between JA and IAA signaling	Ou et al., 2011
ERF091 AT4G18450	ACID	AP2/ERF TF	Ethylene signaling	Ou et al., 2011
ERF095 (ETHYLENE AND SALT INDUCIBLE 1) (AT3G23220)	ACID	AP2/ERF TF	Ethylene ABA signaling, salt tolerance	Ou et al., 2011
ERF098 (AT3G23230)	ACID	AP2/ERF TF	Salt tolerance	Çevik et al., 2012
MYC2 (AT1G32640)	ACID	bHLH TF	JA signaling	Çevik et al., 2012; Chen et al., 2012
MYC3 (AT5G46760)	ACID	bHLH TF	JA signaling	Çevik et al., 2012
MYC4 (AT4G17880)	ACID	bHLH TF	JA signaling	Çevik et al., 2012
JAZ9 (AT1G70700)		Repressor	JA signaling	Zhang et al., 2015
BZS1 (B-BOX DOMAIN PROTEIN 20 (AT4G39070)	ACID	Putative zinc finger transcription factor	Photomorphogenesis, brassinosteroids and light signaling	Ou et al., 2011; Çevik et al., 2012
PHR1-LIKE 1 (PHL1) protein (AT5G29000)	ACID	Homeodomain-like superfamily protein or MYB-CC transcription factor	Phosphate (Pi) and iron starvation; salt stress	Elfving et al., 2011; Ou et al., 2011
CDK8;1 cyclin-dependent kinase 8 (AT5G63610)	nd	Kinase, Mediator complex component	Disease resistance, JA signaling	Zhu et al., 2014
WAX INDUCER1 (WIN1) AT1G15360	nd	AP2/ERF	Biotic and abiotic stress tolerance	Zhu et al., 2014
DEHYDRATION-RESPONSIVE ELEMENT BINDING PROTEIN 2 (DREB2A) (AT5G05410)	ACID	AP2/ERF	Drought tolerance	Elfving et al., 2011; Çevik et al., 2012; Aguilar et al., 2014
VP16-FLOWERING LOCUS C (FLC)-fusion (AT5G10140)	nd	MADS-box protein	Repressor of flowering	Fujiwara et al., 2014
HYPERSENSITIVE TO RED AND BLUE (AT5G49230)	nd	A nuclear localized protein with a ZZ zinc finger domain	Light responses, flowering	Kang et al., 2007
HIGH SUGAR RESPONSE 8 (AT1G30620)	nd	UDP-D-XYLOSE 4-EPIMERASE 1	Cell wall biosynthesis	Seguela-Arnaud et al., 2015
MYB104 (AT2G26950)	ACID	R2R3 factor gene family	Unknown	Çevik et al., 2012
VP16 Herpes simplex virus	ACID	Viral transcriptional activator or TF		Aguilar et al., 2014
WRKY10 (AT1G55600)	ACID	WRKY TF		Çevik et al., 2012
ETHYLENE-INSENSITIVE3 (EIN3) (AT3G20770)	nd	TF	ET signaling; iron homeostasis	Yang et al., 2014
ETHYLENE-INSENSITIVE3-LIKE 1 (EIL1) (AT2G27050)	nd	TF	ET signaling; iron homeostasis	Yang et al., 2014
MEDIATOR16 (MED16) (AT4G04920)	vWF-A domain	Mediator complex subunit	JA and ET signaling, iron homeostasis; disease resistance; cold tolerance	Yang et al., 2014
TaEIL1 (wheat homolog of the Arabidopsis EIL1)	ACID	TF	Ethylene signaling	Liu et al., 2016
AUXIN RESPONSE FACTOR 7 (ARF7) (AT5G20730)	nd	ARF TF	Auxin signaling	Ito et al., 2016
AUXIN RESPONSE FACTOR 9 (ARF9) (AT4G23980)	nd	ARF TF	Auxin signaling	Ito et al., 2016
AUXIN/INDOLE 3-ACETIC ACID 14 Aux/IAA14 (AT4G14550)	nd	Aux/IAA protein family	Auxin signaling, lateral root development	Ito et al., 2016
Zinc finger homeodomain 1 (ZHFD1) (AT1G69600)	ACID	Zinc finger homeodomain transcriptional factor family	Salt stress	Elfving et al., 2011

*Functions are based on TAIR descriptions. nd, not determined*.

### MED25 and Auxin Signaling

More recently, additional roles for the Mediator complex and MED25 in auxin signaling has been identified. It appears that MED13/MAB2/GCT (MACCHI-BOU2/GRAND CENTRAL), which is part of the CDK8 kinase domain of the Mediator complex, regulates auxin signaling through the association with ARF7 and ARF19 and Aux/IAA14 (Ito et al., 2016). Under low auxin levels, Aux/IAA14 represses ARF7 and ARF9 by forming a complex with the co-repressor TPL and the Mediator subunit MED13 to inhibit the interaction between the core Mediator complex and RNA Pol II (**Figure 2**). Under high auxin levels, Aux/IAA14 is degraded and this results in the dissociation of TPL and MED13 from the complex. ARF7 and ARF9, by binding to the auxin responsive element (AuxE) found in the *LBD16* (*LOB DOMAIN 16*) promoter, interact with MED25 to recruit the core Mediator complex and RNA Pol II (Ito et al., 2016).

### MED25: A Shared Component of JA and Auxin Signaling?

Jasmonate and auxin signaling share many mechanistic similarities (reviewed by Pérez and Goossens, 2013). Firstly, similarly to COI1, the F-box protein TIR1 (TRANSPORT INHIBITOR RESPONSE 1) acts as an auxin receptor. Secondly, similarly to the role of JAZ proteins acting as repressors of JA signaling, under low hormone levels, auxin responses are suppressed by Aux/IAA repressors, which recruit the co-repressor TOPLESS (TPL) through their EAR domains. Thirdly, similarly to the activation of JA signaling through the COI1-mediated degradation of JAZ repressors, the reception of auxin leads to the TIR1-mediated degradation of repressors and the activation of the downstream signaling pathway by ARFs (AUXIN RESPONSE FACTORS) acting as positive regulators of auxin signaling through (Kazan and Manners, 2009).

As stated above, MED25 interacts with both activators (MYCs and ERFs) and repressors (JAZs) of JA signaling (Çevik et al., 2012). Therefore, the discovery that MED25 interacts with both activators and repressors of IAA signaling (Ito et al., 2016; **Figure 2**), suggests that MED25 performs mechanistically generic roles in both signaling pathways. Furthermore, as stated above, MED25 interacts with CDK8 during the regulation of JA signaling (Zhu et al., 2014), and with MED13, a component of the CDK8 kinase, during the regulation of auxin signaling (Ito et al., 2016).

### MED25 and Ethylene Signaling

Ethylene signaling is involved in the regulation of biotic and abiotic stresses (Kazan, 2015), including iron (Fe) deficiency in plants (Lucena et al., 2015). The link between MED25 and ethylene signaling became evident when the involvement of MED25 in Fe deficiency responses was discovered (Yang et al., 2014). The *med25* mutant shows increased sensitivity to Fe deficiency, suggesting that MED25 is a positive regulator of these responses. MED25 interacts with both MED16, another Mediator subunit involved in regulating Fe deficiency responses, and EIN3 (ETHYLENE INSENSITIVE 3) and EIL1 (ETHYLENE INSENSITIVE 3 LIKE), known regulators of both ethylene signaling and Fe deficiency responses (Brumbarova et al., 2015). EIN3 and EIL1 regulate the expression of bHLH TF FIT1 (Fe DEFICIENCY INDUCED TF 1), which in turn regulates the expression of Fe-stress responsive genes *IRT1* (*IRON-REGULATED TRANSPORTER 1*) and *FRO2 (FERRIC REDUCTION OXIDASE 2)* (Yang et al., 2014; Zhang et al., 2014).

### MED25 and ABA Signaling

The *med25* mutant shows increased sensitivity to the inhibition of germination and seedling growth by ABA, suggesting a role for MED25 in regulating ABA signaling. In addition, *Em6*, and *RAB-RELATED GENE18* (*RAB18*) show increased ABA responsiveness in the *med25* mutant, suggesting that MED25 is a negative regulator of ABA responses during seed germination and early seedling growth (Chen et al., 2012). Supporting this notion, MED25, physically interacts with ABI5 (ABA-INSENSITIVE5), a basic Leu zipper TF that regulates ABA signaling during seed maturation and germination (Chen et al., 2012). The positive and negative regulation of JA and ABA signaling through interaction with MYC and ERF TFs (Çevik et al., 2012; Chen et al., 2012) and ABI5 (Chen et al., 2012), respectively, also suggests that MED25 may have a role in modulating the JA-ABA crosstalk (Anderson et al., 2004; Liu and Avramova, 2016).

### MED25 Regulates Disease Resistance

Given its role as a positive regulator of JA signaling, a role for MED25 in plant disease resistance is also expected. The JA signaling pathway provides susceptibility to the root infecting pathogen *Fusarium oxysporum* and the bacterial pathogen *Pseudomonas syringae* DC3000 but is required for resistance to leaf infecting necrotrophic pathogens (Thatcher et al., 2009 reviewed by Kazan and Lyons, 2014). Mutant analyses show MED25 differentially regulates resistance to different pathogens. The *med25* mutant shows increased resistance *F. oxysporum* (Kidd et al., 2009) and *P. syringae* (Chen et al., 2012) but increased susceptibility to leaf infecting necrotrophs *A. brassicicola, B. cinerea*, and *Sclerotinia sclerotiorum* (Kidd et al., 2009; Wang et al., 2015). This finding is consistent with the known function of MED25 in JA signaling. There is also evidence that MED25 regulates the interaction of plant roots with soil microbes (Carvalhais et al., 2015).

### MED25 Regulates Abiotic Stress Tolerance

The function of MED25 is not restricted to biotic stress signaling. MED25 physically interacts with abiotic-stress associated TFs, DREB2A, ZFHD1, and MYB-Like (Elfving et al., 2011; **Table 1**). Upon binding of DREB2A to MED25, conformational alterations occur in the overall Mediator structure (Blomberg et al., 2012). The Arabidopsis mutants for these three TF genes as well as the *med25* mutant show increased sensitivity to salt stress, suggesting that MED25 is a positive regulator of salt tolerance. In contrast, the *med25* mutant show increased drought tolerance (Elfving et al., 2011). Therefore, MED25 seems to differentially regulate different abiotic stress responses.

### MED25 as a Regulator of Plant Development

#### Light Signaling and Flowering Time

As stated above, MED25 was first identified as a regulator of flowering time under shade conditions and was placed downstream from phyB (Cerdán and Chory, 2003). However, the regulation of flowering time by MED25 seems to be complex and involve multiple components and mechanisms (Wollenberg et al., 2008) and currently very little is known how phyB and other photoreceptors regulate MED25-mediated responses. For instance, subsequent studies have suggested that MED25 modulates flowering through both CONSTANS-dependent and independent pathways (Iñigo et al., 2012a). Another study suggested that MED25 performs different roles during photomorphogenesis and flowering. As a positive regulator of flowering, MED25 seems to be acting downstream from phyA to promote the expression of *FT*, which positively regulates flowering by suppressing *FLC*, a negative regulator of flowering (Klose et al., 2012). In addition, MED25 positively regulates CONSTANS (CO), a positive regulator of *FT*, to promote flowering. The regulatory effect of MED25 on photomorphogenesis seems to be achieved through HY5, a positive regulator of this process (Klose et al., 2012; Koprivova et al., 2014). The RING-H2 proteins MBR1 and MBR2 (MED25-BINDING RING-H2 PROTEIN1 and 2) interact and degrade MED25 are also involved in the modulation of flowering (**Table 1**). Interestingly, the ubiquitination of MED25 by MBR1 and MBR2 was linked to the activation of *FT* transcription in a process called “activation by destruction” (Iñigo et al., 2012b).

Remarkably, the short tandem repeat section found in the C-terminus of MED25 (**Figure 1**) seems to have a role in modulating flowering time. This section contains encodes a poly-glutamine track and transgenic plants expressing a MED25 isoform lacking this domain show similar flowering phenotypes as the *pft1* mutant (Rival et al., 2014). It should be noted that the alternative transcription of MED25 affects this region and the shorter MED25 isoform is predicted to be lacking vWDF-A required for binding of MED25 to the Mediator complex. Therefore, it is possible that the functions performed by shorter MED25 isoform are independent from the Mediator complex.

#### Reactive Oxygen Signaling and Root Development

A role for MED25 in negative regulation of auxin (IAA) signaling as well as primary and lateral root formation and development has been shown (see below) (Raya-González et al., 2014). The *med25* mutant has shorter root hairs than wild type and this phenotype could be rescued by exogenous application of ROS (H_2_O_2_), suggesting that MED25-mediated changes in redox status regulate root hair differentiation. MED25 positively regulates class III peroxidases involved in H_2_O_2_ production while negatively regulating NADPH oxidases involved in ROS generation. This changes the redox status of the cells, which then acts as a signal in the regulation of cell specification, initiation and elongation of root hairs (Sundaravelpandian et al., 2013). In addition, MED25 negatively regulates primary and lateral root growth by negatively regulating the expression of genes such as *PIN1* and *PIN2* involved in auxin transport and response required for meristematic cell proliferation at the root tips (Raya-González et al., 2014).

#### Final Organ Size

The *med25* mutant has larger floral organs (sepals, petals, stamens, etc.), suggesting that MED25 limits organ growth most likely by negative regulation of genes that encode positive regulators of cell expansion and proliferation (Xu and Li, 2011). This phenotype was explained by higher expression of expansin-encoding genes in the *med25* mutant (Xu and Li, 2011).

#### Cell Wall and Elongation

The *soh715* mutation, which acts as a repressor of the sugar hypersensitive phenotype of the *hsr8-1* mutant, was found to be allelic to *med25* (Seguela-Arnaud et al., 2015). The reduced hypocotyl elongation phenotype of *hsr8-1* is suppressed by the *med25* mutation independently from phytochrome and JA pathways. Subsequent studies show that MED25 is required for the expression of glucose-induced genes that encode proteins involved in cell wall, anthocyanin and flavonoid and glucosinolate pathways (Seguela-Arnaud et al., 2015).

### MED25 Function Is Conserved between Monocots and Dicots

So far, the function of MED25 has been mainly investigated in Arabidopsis. However, emerging evidence indicates that at least some MED25-mediated functions are conserved between dicots and monocots. For instance, TaMED25, the wheat ortholog of MED25, complements the defense and developmental phenotypes of the Arabidopsis *med25* mutant (Kidd et al., 2009), suggesting that TaMED25 performs functionally similar roles to MED25. Wheat plants with one or two subgenome copies or homoeologs of deleted *TaMED25* were successfully isolated from a heavy ion bombarded hexaploid wheat population (Fitzgerald et al., 2010). However, despite extensive efforts, complete knockouts with deleted A, B, and D homoeologs of TaMED25 could not be isolated. This seems to suggest an essential function for TaMED25 in wheat (Fitzgerald et al., 2015). However, wheat and barley plants with reduced expression of *TaMED25* and *HvMED25* by virus-induced gene silencing show increased resistance to the powdery mildew pathogen, *Blumeria graminis* f. sp. *tritici* (Liu et al., 2016). Therefore, similarly to the Arabidopsis MED25 conferring susceptibility to *F. oxysporum* and *P. syringae*, TaMED25 acts as a powdery mildew susceptibility gene in wheat and barley.

Moreover, similarly to Arabidopsis MED25, which interacts with EIN/EIL1, TaMED25 interacts with TaEIL1, the wheat homolog of Arabidopsis EIL1 (Liu et al., 2016). Although, if TaMED25 and TaEIL1 are involved in regulating Fe-deficiency responses is unknown, the interaction between TaMED25 and TaEIL1 certainly supports a role for TaMED25 in ethylene signaling in wheat. Future studies may reveal additional MED25-regulated functions that are conserved in plants.

### Is MED25 the Only Multifunctional Mediator Complex Subunit in Plants

As reviewed here, it is becoming evident that MED25 is master regulator of transcriptional responses to multiple endogenous and exogenous signals (Balderas-Hernández et al., 2013). Given that there are 1000s of TFs in plants but only a limited number of Mediator subunits, particularly those that reside in the TF-interacting Tail module of the Mediator complex, it is probably not surprising that many different TFs may interact with the same Mediator subunit to initiate gene expression. For this reason, different Mediator subunits seem to perform both diverse and overlapping roles. For instance, MED16, a Tail module subunit, regulates SA and JA signaling (Wathugala et al., 2012), Fe homeostasis (Yang et al., 2014), cold-acclimation (Hemsley et al., 2014) and regulation of transcriptional responses to cell wall defects (Sorek et al., 2015). The Middle module subunit MED8 regulates flowering time (Kidd et al., 2009), cell wall development (Seguela-Arnaud et al., 2015) as well as defense (Kidd et al., 2009; An and Mou, 2013). MED18, located in the Head module of the Mediator complex, regulates flower development (Zheng et al., 2013) and plant defense (Lai et al., 2014; Liao et al., 2016; Fallath et al., 2017).

In addition, a few other Mediator complex subunits seem to regulate overlapping functions as MED25. For instance, similarly to MED25, MED16 recruits RNA polymerase II to the promoters *ORA59* and *PDF1.2*. MED16 interacts with WRKY33, a defense associated TF and is required for pathogen (*B. cinerea*)-mediated induction of *PDF1.2* (Wang et al., 2015, 2016). However, in contrast to MED25, which is required for both insect/wound and pathogen response branches of the JA signaling pathway, MED16 seems to be specific for pathogen defense (Wang et al., 2015). Given that the expression of *ORA59*, *ERF1* and *PDF1.2*, are influenced by both JA and ethylene pathways, it is tempting to speculate that MED25 is mostly involved in JA whereas MED16 is in the ET pathway. Similarly to MED25, MED8 regulates disease resistance, flowering time (Kidd et al., 2009) and organ size (Xu and Li, 2012).

## Conclusion

**FIGURE 3:**
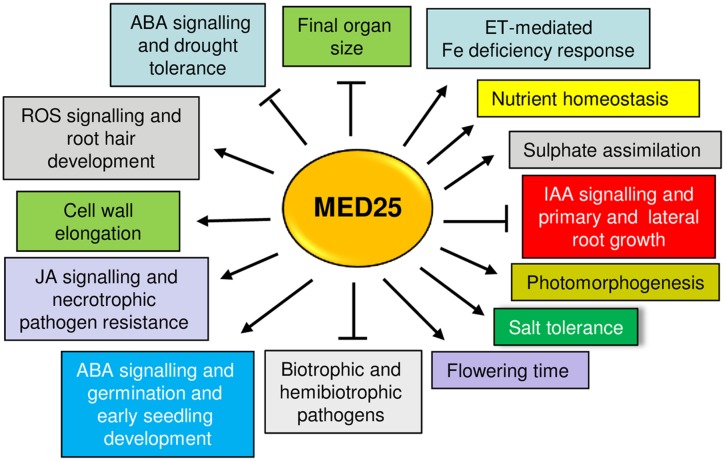
An overview of different plant functions positively (arrows) or negatively (blunt arrows) regulated by MED25. Please see text for details.

The studies reviewed here show that MED25 is multifunctional protein (**Figure 3**). Although MED25 and other Mediator subunits play essential roles in plant signaling, abolishing the function of a single Mediator subunit often does not result in complete loss of transcription, suggesting that engineering of Mediator subunits can be a potential strategy to improve stress tolerance in plants. The findings reviewed here might imply that the engineering of MED25 can potentially lead to pleiotropic effects due to its involvement in multiple plant functions. However, the ability of MED25 to perform different roles through its different domains can offer a flexibility in this regard. For instance, the modification of the MED25 ACID region through technologies such as CRISPR/CAS9 (Clustered regularly interspaced short palindromic repeats/CRISPR-associated protein-9) and may allow its interaction only with certain TFs while abolishing or attenuating interactions with others. Alternatively, new specificities can be generated by adding the ACID binding ability to heterologously-expressed TFs. Given that VP16 is a strong transcriptional activator and binds to the MED25 ACID region (Yang et al., 2004) various activators or repressors could be expressed as VP16 fusion proteins to alter MED25-regulated transcription (Fujiwara et al., 2014). The identification of small molecules that bind to the ACID region and can either activate or inhibit transcription by modulating the TF binding to this region may be a useful strategy. This strategy has recently been shown for the ABA receptor PYRABACTIN RESISTANCE 1 (PYR1), which can be activated by small molecules mimicking ABA action (Park et al., 2015). Better understanding the potential roles played by MED25 and the Mediator complex advances our understanding of transcriptional regulation in plants while providing opportunities to modify plant development and improve stress tolerance.

## Notes

After the acceptance of this article, two papers published have revealed additional insights into PFT1/MED25 functions in flowering and root development. Liu et al. (2017) showed that the microRNA319-regulated TEOSINTE BRANCHED/CYCLOIDEA/PCF (TCP) transcription factors and the flowering activators FLOWERING BHLH (FBHs) physically interact with PFT1/MED25 to promote CO transcription during flowering. A second paper by Muñoz-Parra et al. (2017) reported that PFT1/MED25 is a regulator of high density-modulated root traits.

## Author Contributions

KK reviewed the literature and wrote the paper.

## Conflict of Interest Statement

The author declares that the research was conducted in the absence of any commercial or financial relationships that could be construed as a potential conflict of interest.
